# Defining the Impact of Non-Native Species

**DOI:** 10.1111/cobi.12299

**Published:** 2014-04-29

**Authors:** Jonathan M Jeschke, Sven Bacher, Tim M Blackburn, Jaimie T A Dick, Franz Essl, Thomas Evans, Mirijam Gaertner, Philip E Hulme, Ingolf Kühn, Agata Mrugała, Jan Pergl, Petr Pyšek, Wolfgang Rabitsch, Anthony Ricciardi, David M Richardson, Agnieszka Sendek, Montserrat VilÀ, Marten Winter, Sabrina Kumschick

**Affiliations:** * Department of Ecology and Ecosystem Management, Restoration Ecology, Technische Universität München85350, Freising-Weihenstephan, Germany; † Unit Ecology & Evolution, Department of Biology, University of FribourgChemin du Musée 10, 1700, Fribourg, Switzerland; ‡ Institute of Zoology, Zoological Society of LondonRegent's Park, NW1 4RY, London, United Kingdom; § Distinguished Scientist Fellowship Program, King Saud UniversityRiyadh, 1145, Saudi Arabia; ** Institute for Global Food Security, School of Biological Sciences, Queen's University BelfastM.B.C., 97 Lisburn Road, Belfast, BT9 7BL, Northern Ireland, United Kingdom; †† Department of Conservation Biology, Vegetation and Landscape Ecology, University of ViennaRennweg 14, 1030, Vienna, Austria; ‡‡ Silwood Park Campus, Department of Life Sciences, Imperial College LondonBuckhurst Road, Ascot, Berkshire, SL5 7PY, United Kingdom; §§ Centre for Invasion Biology, Department of Botany and Zoology, Stellenbosch UniversityPrivate Bag X1, Matieland, 7602, South Africa; *** The Bio-Protection Research Centre, Lincoln UniversityPO Box 84, Christchurch, New Zealand; ††† Department of Community Ecology, UFZ, Helmholtz Centre for Environmental Research – UFZTheodor-Lieser-Str. 4, 06120, Halle, Germany; ‡‡‡ German Centre for Integrative Biodiversity Research (iDiv) Halle-Jena-LeipzigDeutscher Platz 5e, 04103, Leipzig, Germany; §§§ Department of Ecology, Faculty of Science, Charles University in PragueViničná 7, CZ-128 44 Praha 2, Czech Republic; **** Institute of Botany, Academy of Sciences of the Czech RepublicCZ-252 43, Průhonice, Czech Republic; †††† Environment Agency Austria, Department of Biodiversity and Nature ConservationSpittelauer Lände 5, 1090, Vienna, Austria; ‡‡‡‡ Redpath Museum, McGill University859 Sherbrooke Street West, Montreal, Quebec, Canada, H3A0C4; §§§§ Estación Biológica de Doñana (EBD-CSIC), Avda. Américo Vespucios/n, Isla de la Cartuja, 41092, Sevilla, Spain; ***** Institute of Biology/Geobotany and Botanical Garden, Martin-Luther-University Halle-WittenbergAm Kirchtor 1, 06108 Halle, Germany

**Keywords:** biological invasions, definitions, ecological and socio-economic impacts, exotic species, human perception, invasion biology, invasive alien species, biología de la invasión, especies exóticas, especies foráneas invasoras, definiciones, impactos ecológicos y socio-económicos, invasiones biológicas, percepción humana

## Abstract

Non-native species cause changes in the ecosystems to which they are introduced. These changes, or some of them, are usually termed impacts; they can be manifold and potentially damaging to ecosystems and biodiversity. However, the impacts of most non-native species are poorly understood, and a synthesis of available information is being hindered because authors often do not clearly define impact. We argue that explicitly defining the impact of non-native species will promote progress toward a better understanding of the implications of changes to biodiversity and ecosystems caused by non-native species; help disentangle which aspects of scientific debates about non-native species are due to disparate definitions and which represent true scientific discord; and improve communication between scientists from different research disciplines and between scientists, managers, and policy makers. For these reasons and based on examples from the literature, we devised seven key questions that fall into 4 categories: directionality, classification and measurement, ecological or socio-economic changes, and scale. These questions should help in formulating clear and practical definitions of impact to suit specific scientific, stakeholder, or legislative contexts.

Definiendo el Impacto de las Especies No-Nativas

**Resumen:**

Las especies no-nativas pueden causar cambios en los ecosistemas donde son introducidas. Estos cambios, o algunos de ellos, usualmente se denominan como *impactos*; estos pueden ser variados y potencialmente dañinos para los ecosistemas y la biodiversidad. Sin embargo, los impactos de la mayoría de las especies no-nativas están pobremente entendidos y una síntesis de información disponible se ve obstaculizada porque los autores continuamente no definen claramente *impacto*. Discutimos que definir explícitamente el impacto de las especies no-nativas promoverá el progreso hacia un mejor entendimiento de las implicaciones de los cambios a la biodiversidad y los ecosistemas causados por especies no-nativas; ayudar a entender cuáles aspectos de los debates científicos sobre especies no-nativas son debidos a definiciones diversas y cuáles representan un verdadero desacuerdo científico; y mejorar la comunicación entre científicos de diferentes disciplinas y entre científicos, administradores y quienes hacen las políticas. Por estas razones y basándonos en ejemplos tomados de la literatura, concebimos siete preguntas clave que caen en cuatro categorías: direccionalidad, clasificación y medida, cambios ecológicos o socio-económicos, y escala. Estas preguntas deberían ayudar en la formulación de definiciones claras y prácticas del impacto para encajar mejor con contextos científicos, de las partes interesadas o legislativos específicos.

## A Call to Define the Impact of Non-Native Species

The introduction of species beyond their native range as a direct or indirect result of human action (termed *non-native species* here) causes changes in the ecosystems to which they are introduced. In some cases, these changes are dramatic and may result in the extinction of native species or radical changes in ecosystem functioning, but for the vast majority of non-native species no quantitative information is available on the consequences of such introductions (Kulhanek et al. 2011; Larson et al. 2013; Simberloff et al. 2013). We do know that the impacts of non-native species generally increase if the species establish themselves and spread in their new environment (i.e., if they become invasive sensu Blackburn et al. [2011]), but non-native species can have impacts even when they are not established or widespread (Ricciardi & Cohen 2007; Jeschke et al. 2013; Ricciardi et al. 2013). Indeed, non-native species can have impacts as soon as they are introduced; for example, pathogens can affect the health of animals, plants, or other organisms immediately after their arrival in the new environment. The breadth and potential severity of the impacts of non-native species means that a better understanding of them is of broad relevance, for example, for prioritizing management, conservation and restoration actions, and for appropriate policy responses to invasions.

Our collective experience is that progress toward this understanding is being hindered because authors often do not explicitly or clearly define the impacts of non-native species. The current literature on impacts is complicated by a plethora of different approaches to their quantification that are associated with a concomitant range of impact metrics (Hulme et al. 2013). We argue that if authors are routinely explicit about their definition of impacts of non-native species, it will be possible to synthesize the growing body of work on this topic more effectively. For instance, systematic reviews, comparative analyses, and meta-analyses (Koricheva et al. 2013) can be much more informative if the authors of studies included in such assessments clearly define impact and clearly explain how impact was measured. If authors do not, the synthesis of available data can become difficult or even impossible.

Explicit definitions of impact will also help disentangle which aspects of scientific debates about non-native species (see e.g., Gurevitch & Padilla 2004; Ricciardi 2004; Davis et al. 2011; Simberloff et al. 2011) are due to disparate definitions (including spatio-temporal scale, taxonomic focus, and consideration of human values) and which represent true scientific discord (i.e., a difference of opinion on a mutually understood argument, rather than on disjunct arguments). Distinguishing between these will help identify questions that should be research priorities.

A third reason for explicitly defining the impact of non-native species is that communication between scientists from different research disciplines and between scientists and stakeholders (e.g., managers, conservationists, and policy makers) will improve if clarity regarding the meaning of *impact* can be achieved. Decision science applies a clarity test to overcome the problem of different people assigning different meanings to the same term (see Howard [1988] for details). To pass a clarity test, *impact* must be explicitly and unambiguously defined. An area where clarity for improved communication is particularly relevant is the regulatory assessment of risks posed by non-native species (e.g., FAO 2004; EPPO 2007; EFSA 2011). In these assessments, experts from different sectors are typically involved, and they often have divergent views on how *impact* should be defined (Boonman-Berson et al. 2014).

For these 3 reasons—promoting progress toward a better understanding of impacts, discriminating between disparate definitions and scientific discord, and improving communication—we recommend that *impact* in the context of non-native species be explicitly and clearly defined in scientific publications, stakeholder discussions, and other important contexts such as legislation. We formulated a set of questions to inform this exercise (see Heger et al. [2013] for questions that help define *alien* [i.e., *non-native*] and *invasive species*). Because different definitions can be suitable for different purposes, we do not call for a universal definition of impacts, but rather for explicit and clear definitions that reflect their particular context and audience.

## Key Questions to Help Define the Impact of Non-Native Species

**Figure 1:**
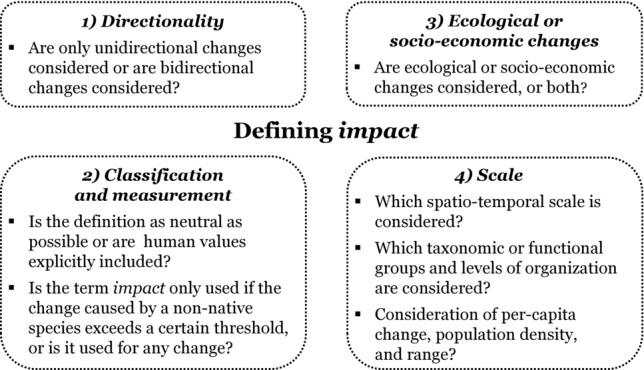
Questions that help define the impact of non-native species, organized into 4 categories: directionality, classification and measurement, ecological or socio-economic changes, and scale.

Questions that may serve as guidance to define the impact of non-native species fall into 4 categories: directionality, classification and measurement, ecological or socio-economic changes, and scale (Fig.1). Many of the questions include the term *change*, reflecting the fact that the impacts of non-native species are due to changes caused by them. Such changes may occur proximally (i.e., within the regions or system in which they are introduced) or distally (e.g., downstream of the population of a non-native species that has changed water runoff or sedimentation rates [Zedler & Kercher 2004])—even over substantial distances (e.g., effects of allergenic pollen of non-native plants [Šikoparija et al. 2013]).

### Directionality

Are only unidirectional changes considered (e.g., potential decrease in species diversity), or are bidirectional changes considered (e.g., potential increase or decrease in species diversity)? For example, Goodenough (2010), Schlaepfer et al. (2011), and Kumschick et al. (2012) looked at bidirectional changes caused by non-native species, whereas Olenin et al. (2007), Nentwig et al. (2010), and the international organizations FAO (2004), EPPO (2007), and EFSA (2011) considered only unidirectional changes. The latter makes sense for risk analyses, which typically focus on the potential for deleterious impacts of non-native species, whereas cost-benefit or multicriteria analyses (reviewed by Dana et al. [2014]) demand consideration of bidirectional changes (deleterious and beneficial impacts). Also, considering bidirectional changes may better capture the complexity of ecosystem dynamics. For instance, Pyšek et al. (2012) showed that plant species richness and measures of plant community structure tend to decrease following invasion, whereas the abundance and richness of the soil biota—as well as concentrations of soil nutrients and water—more often increase than decrease following invasion.

### Classification and Measurement

Are impacts classified and quantified as neutrally as possible (e.g., solely based on the direction and magnitude of change), or are human values explicitly included? Daehler (2001), Rejmánek et al. (2002), Ricciardi et al. (2013), and Simberloff et al. (2013) define impacts neutrally. One challenge for a neutral definition is whether human values can (or indeed should) be excluded altogether. Larson et al. (2013) argue that the term *impact* is already value laden, and a more neutral term might be *change* or *effect*. Other authors define *impact* by explicitly including human values (e.g., Davis & Thompson 2000, 2001), and international regulatory bodies and organizations routinely do so (FAO 2004; EPPO 2007; EFSA 2011; IUCN 2013).

If definitions include human values, we suggest 2 components of an impact be discriminated: first, magnitude of change, which is neutrally quantifiable and, second, the value of the change assigned by humans (Kumschick et al. 2012). A challenge, however, is that the change may be perceived as valuable to one part of society but detrimental to another (e.g., Kumschick et al. 2012; Heger et al. 2013; Simberloff et al. 2013). For example, the presence of non-native fish and game species may be valued by anglers and hunters, but conservationists may perceive the same species as a threat to native biodiversity. Even economic stakeholders may have diametrically opposing views of non-native species, as in New Zealand, where non-native Scotch broom (*Cytisus scoparius*) is seen as valuable by beekeepers, while farmers and forestry industry people opt for releasing biocontrol agents against this species (Jarvis et al. 2006).

Keeney (1992) outlines how value-focused thinking can improve decision making. This approach may be useful in the context of non-native species. For example, the approach can be applied to classify changes caused by non-native species as either decision relevant or decision irrelevant. If stakeholders have different values, they will find different changes to be decision relevant and may differ in their views on the benefits of such changes (as illustrated by examples above). The application of decision science to this process can help managers and policy makers reach decisions despite conflicting viewpoints, although an exploration of this approach in practice is beyond the scope of this current article.

Is the term *impact* used only when the change caused by the non-native species exceeds a certain threshold, or is it used for any change? Ricciardi et al. (2013) define impact as a measurable change (recognizing detection thresholds), whereas Hulme et al. (2013) and Simberloff et al. (2013) define impact as a significant change (here, statistical significance should be discriminated against other types of significance, particularly biological significance). Thresholds of impact are potentially important because they relate to the magnitude and potential reversibility of different changes. Some non-native species (i.e., *transformers* [Richardson et al. 2000]) can induce regime shifts and modify ecosystem functioning, enhancing their own abundance and persistence, and suppressing those of native species through modification of feedback processes (Nicholls et al. 2011; Seastedt & Pyšek 2011). Martin et al. (2009) outline a structured decision making framework for considering thresholds in the context of conservation and management that could be applied to impacts of non-native species.

### Ecological or Socio-Economic Changes

Are ecological or socio-economic changes, or both, considered? Many studies (e.g., Pyšek et al. 2012; Ricciardi et al. 2013) have focused on ecological changes such as changes in population densities or ranges of native species, whereas other studies (e.g., Nentwig et al. 2010; Kumschick et al. 2012) also considered socio-economic changes such as those in agriculture, animal production, forestry, infrastructure, or human well-being. Although ecological and socio-economic impacts appear to be correlated (Vilà et al. 2010), the relationship between them needs to be better investigated: A strong ecological impact (e.g., extinction of a species) is not necessarily connected with a strong socio-economic impact.

### Scale

Which spatial (e.g., local, regional, national, continental, global; or islands only) and temporal (e.g., intermittent, seasonal, transient, and permanent) scales are considered? The focal scale has a huge influence on almost any statement about impact. For instance, the introduction of non-native species can lead to net increases in species richness at small spatial scales (e.g., where fewer species, if any, become extirpated than are introduced) and cause a decline in global species richness through the extinction of endemic or locally rare native species (Sax & Gaines 2003; Clavero & García-Berthou 2005). Also, there can be large differences between the short- and long-term impacts of non-native species (Strayer et al. 2006). Results of a meta-analysis on declines of native species attributable to biological invasions in Mediterranean ecosystems showed that studies conducted at small scales or sampled over long periods reveal stronger impacts of non-native species than those conducted at large spatial scales and over short periods (Gaertner et al. 2009). The inclusion or exclusion of predicted future impacts of a non-native species should also be made explicit by authors (as is done by FAO 2004; EPPO 2007; and EFSA 2011). If the non-native species is still rare but rapidly spreading, currently documented impacts will typically be small, whereas anticipated future impacts (predicted from experiments or impacts caused in other regions invaded earlier or from species traits or high-impact congeners) may be much larger.

Which taxonomic or functional groups (e.g., animals, plants, fungi, parasites, parasitoids, viruses, or other pathogens) and levels of organization (e.g., genetic, population, species, community, ecosystem, and landscape) are considered? For example, Vilà et al. (2011) demonstrated that by the time changes in ecosystem processes (e.g., nutrient cycling) due to non-native species are detected, major impacts on plant species and communities are likely to have already occurred. In another study, Vilà et al. (2010) found taxonomic differences in the proportion of non-native species with known ecological and economic impacts in Europe. Sax (2002) provided a multitaxon analysis of invader impacts in Californian woodlands. In general, however, cross-taxonomic studies are rare in invasion ecology (Jeschke et al. 2012), and it would be useful for more studies to investigate impacts of non-native species across taxa and functional groups (see also Sax et al. 2005). Such studies are vital for a general understanding of impacts.

Is change considered per capita or per biomass of the non-native species; locally for the non-native population (per capita change × population density); or over the full range of the non-native species (per capita change × population density × population range [cf. Parker et al. 1999])? For example, the impact scoring system of Kumschick and Nentwig (2010) and Nentwig et al. (2010) defines impact in 2 different ways: potential impact includes per capita impact and abundance, whereas actual impact additionally factors in the extent of the occupied range; species can rank high on potential but not actual impact or vice versa.

## Resolving Disparity through Greater Clarity

These questions highlight considerations that may resolve substantial confusion about the impact of non-native species. They allow all—researchers, managers, policy makers, and others—who use the term *impact* to explicitly and clearly define it. In this way, progress toward a better understanding of impacts will be promoted, particularly because a synthesis of available information and data can be more informative. Aside from a suitable definition of *impact*, meta-analyses and other quantitative approaches for synthesis depend on studies that adequately measure impact. How impact should be measured depends on how it is defined. For example, if one is only interested in economic changes caused by non-native species (Are ecological or socio-economic changes, or both, considered?), impacts could be adequately measured in monetary terms. If ecological changes should be considered as well, a comprehensive impact score might be more adequate (see Kumschick & Nentwig [2010] and Nentwig et al. [2010] for examples of such scoring systems). Guidance on the choice of metrics is again provided by decision science (e.g., Keeney & Gregory 2005).

Aside from promoting progress toward a better understanding of impacts, explicit and clear definitions of *impact* will, as outlined above, also help one discriminate between disparate definitions and scientific discord and improve communication between scientists from different research disciplines and generally among scientists, managers, and policy makers. Regarding the latter, in our review of the literature on *impact* definitions, we found that many scientific studies quantify impact rather narrowly (e.g., restricted to unidirectional changes, ecological changes, and a limited scale [see above for references]). Yet, what is typically needed for appropriate management and policy actions is an understanding of *impact* in a broader sense. Indeed, regulatory bodies such as FAO (2004), EPPO (2007), or EFSA (2011) typically define the impact of non-native species rather broadly (although they are restricted to unidirectional changes if they follow a risk-assessment approach rather than a cost-benefit or multicriteria framework [Dana et al. 2014]). Explicit definitions of *impact* clearly expose this gap between what is needed by managers and policy makers and what scientists currently deliver. Scientists should be clear about the audience to which their assessment of impacts is directed and ensure their definition is appropriate for guiding subsequent action.
